# Simple, rapid, and accurate malaria diagnostic platform using microfluidic-based immunoassay of *Plasmodium falciparum* lactate dehydrogenase

**DOI:** 10.1186/s40580-020-00223-w

**Published:** 2020-04-11

**Authors:** Wang Sik Lee, Taejoon Kang, Kyung Jin Kwak, Kyoungsook Park, So Yeon Yi, Ui Jin Lee, Yong Beom Shin, Jinyoung Jeong

**Affiliations:** 1 Environmental Disease Research Center, Korea Research Institute of Bioscience and Biotechnology (KRIBB), 125 Gwahak-ro, Yuseong-gu, Daejeon, 34141 Republic of Korea; 2 Department of Nanobiotechnology, KRIBB School of Biotechnology, University of Science and Technology (UST), 217 Gajeong-ro, Yuseong-gu, Daejeon, 34113 Republic of Korea; 3 Bionanotechnology Research Center, Korea Research Institute of Bioscience and Biotechnology (KRIBB), 125 Gwahak-ro, Yuseong-gu, Daejeon, 34141 Republic of Korea; 4 Dept. of General Education, Daejeon Health Institute of Technology, 21 Chungjeon-ro, Dong-gu, Daejeon, 34504 Republic of Korea; 5 BioNano Health Guard Research Center, 125 Gwahak-ro, Yuseong-gu, Daejeon, 34141 Republic of Korea; 6 Department of Biochemistry, College of Natural Sciences, Chungnam National University, 99 Daehak-ro, Yuseong-gu, Daejeon, 34134 Republic of Korea

**Keywords:** Microfluidic microplate, Immunoassay, Malaria, *Pf*LDH, Diagnosis

## Abstract

This work reports on a rapid diagnostic platform for the detection of *Plasmodium falciparum* lactate dehydrogenase (*Pf*LDH), a representative malaria biomarker, using a microfluidic microplate-based immunoassay. In this study, the microfluidic microplate made it possible to diagnose *Pf*LDH with a small volume of sample (only 5 μL) and short time (< 90 min) compared to conventional immunoassays such as enzyme-linked immunosorbent assay (ELISA). Moreover, the diagnostic performance of *Pf*LDH showed high sensitivity, specificity, and selectivity (i.e., 0.025 pg/μL in phosphate-buffered saline and 1 pg/μL in human serum). The microfluidic-based microplate sensing platform has the potential to adapt simple, rapid, and accurate diagnoses to the practical detection of malaria.

## Introduction

Malaria is a serious infectious disease that is transmitted from mosquitoes to humans causing 219 million infections and 435,000 deaths worldwide in 2017 according to the World Health Organization (WHO) [1, 2]. Despite vast efforts to reduce the risk of malaria, it still has high mortality and morbidity due to inaccurate diagnosis and increased drug resistance [3]. Malaria infection is caused by *Plasmodium* parasites that are transmitted by the bite of *Anopheles* spp. mosquitoes such as *Plasmodium falciparum (P. falciparum)* and *Plasmodium vivax* (*P. vivax*) [1]. In particular, *P. falciparum* is an important target of malaria diagnosis because it accounts for 90% of worldwide malaria mortality [4]. *P. falciparum* has various biomarkers including lactate dehydrogenase (LDH), histidine rich protein2 (HRP2), aldolase, and hypoxanthine phosphoribosyl transferase [5]. Conventional diagnosis of *P. falciparum* is mainly dependent on HRP2 detection by immunoassay [6]. However, HRP2 deletion mutants have been reported in several countries [7–12]. Therefore, it is necessary to develop an alternative diagnostic biomarker instead of HRP2. In this study, we chose *P. falciparum* lactate dehydrogenase (*Pf*LDH) as an alternative biomarker for the diagnosis of malaria (*P. falciparum*), which is a water-soluble enzyme that converts pyruvate to lactate in glycolysis in *P. falciparum* infection [5, 13, 14].

Conventional malaria diagnosis methods include a microscopic examination and antibody-based rapid diagnostic tests (RDTs) [5, 15–19]. Although the microscopic examination is the gold standard for malaria diagnosis and is rapid and cost-effective, it requires highly trained personnel. Meanwhile, the RDT, a lateral flow immunoassay, is a useful method in low-resource environments because it is possible to provide cost-effective, rapid (< 30 min) and simple detection methods using the nitrocellulose strip [5, 20]. However, RDTs have sensitivity limitations, such as the inability to detect < 200 parasites/µL or < 1 ng/mL [16, 19, 21, 22]. To overcome the limitations of existing diagnostics, several studies demonstrated improved detection efficiency by introducing nanoparticles such as magnetic beads. Markwalter et al. successfully detected *Pf*LDH up to 21.1 ± 0.4 parasites/mL within 45 min using antibody-immobilized magnetic beads as a colorimetric assay [14]. Additionally, they developed a simultaneous capture and sequential detection of two malarial biomarkers (*Pf*LDH and HRP2) on magnetic microparticles [23]. Kim et al. detected HRP2 up to 0.1 ng/mL using antibody-immobilized magnetic beads and quantum dots using an automated droplet-based microfluidic device [20]. Although these studies were developed to overcome the limitations of conventional enzyme-linked immunosorbent assay (ELISA), nanoparticle-based immunoassays still require a relatively complex surface functionalization process and a large amount of antibody to immobilize the antibody.

To overcome these limitations of conventional ELISA-based diagnosis and to improve the diagnosis of *Pf*LDH, we present a microfluidic microplate-based immunoassay. This microfluidic microplate is an Optimiser™ microplate, which is one of the next-generation immunoassay platforms, and the existing 96-well plate was developed in the form of a microfluidic channel [24–26]. In this study, we demonstrated that the microfluidic microplate-based immunoassay provides an ultrafast, simple, and precise immunoassay for *Pf*LDH diagnosis. The microfluidic microplate-based immunoassay significantly reduced the amount of reagents (5 μL) and diagnosis time (< 90 min) compared to conventional ELISA, as well as enabling high-sensitivity diagnostics (0.025 pg/µL). Additionally, we confirmed that *Pf*LDH in human serum can be diagnosed up to 1 pg/µL. Based on the results of our study, we expect that the microfluidic microplate-based immunoassay platform will be widely used for infectious disease diagnosis as well as in malaria.

## Results and discussion

### Microfluidic microplate-based immunoassay for *Pf*LDH diagnosis

**Fig. 1:**
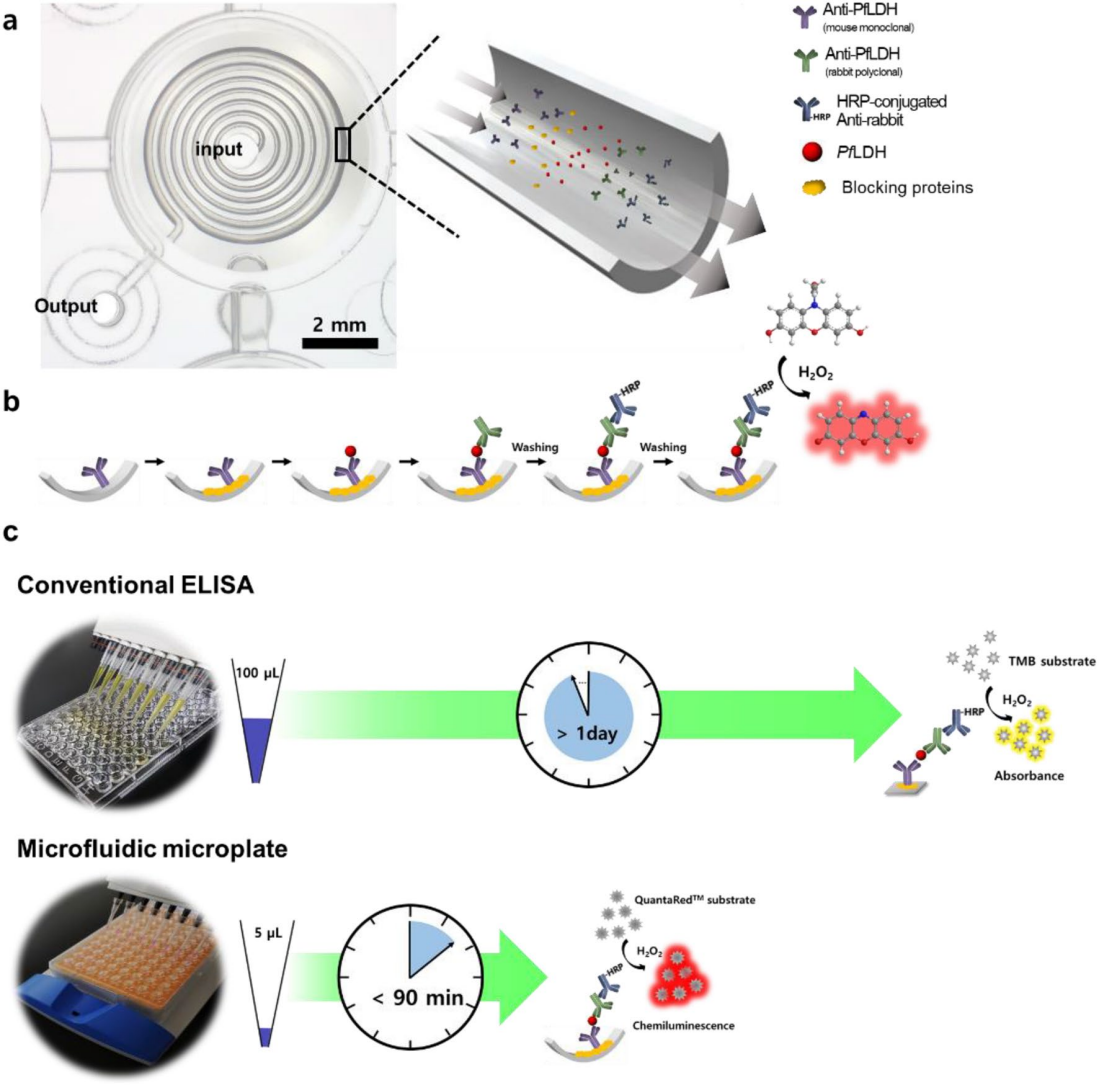
Schematic illustration for diagnosis of *Pf*LDH using microfluidic microplate. **a** Optical image of the microfluidic microplate. The microfluidic microplate allows for low volume and rapid immunoassay due to microfluidic channel. **b** Flow sequence for detection of *Pf*LDH. **c** Comparison of *Pf*LDH detection method using conventional ELISA and microfluidic microplate

Figure 1a illustrates the construction of a microfluidic microplate and the procedure for detecting *Pf*LDH. The whole body of this microplate consisted of a conventional 96-well plate with an inlet for pipette injection, an outlet that is open toward the absorbent pad, and a microfluidic channel between them. The microfluidic microplate is a spiral microfluidic channel and has a 1.5-fold larger surface area and a 50-fold surface-area-to-volume ratio than conventional ELISA plates [24]. The microfluidic microplate is operated by capillary action between the microchannel and adsorbent pad. The process is passive flow regulation, which can lead to accurate and rapid immunoassay results. This flow system involves the sequential addition of reagents, such as antibodies and antigens, to the microfluidic channel (Fig. 1b). The diagnostic procedure in the microfluidic channel is similar to that of conventional ELISA. In general, conventional ELISA was used to target the absorbance signal from the 3,3′,5,5′-tetramethylbenzidine (TMB) substrate; however, the microfluidic microplate-based immunoassay used the chemiluminescence signal from the 10-acetyl-3,7-dihydroxyphenoxazine (ADHP)-based chemiluminescent substrate due to the microfluidic channel structural properties. ADHP is not normally a fluorescent molecule; instead, it is converted to a fluorescent form (resorufin) in the presence of horseradish peroxidase (HRP) and hydroxide peroxide [27]. Consequently, microfluidic microplates have the advantage of using a highly accessible microfluidic surface, capillary design, and highly sensitive substrate compared to ELISA. Therefore, the volume required to diagnose the targets can be significantly reduced, and the overall diagnosis time can be much faster than that of conventional ELISA (Fig. 1c). This immunoassay platform shows that it is appropriate to diagnose *Pf*LDH with a small amount of antibody and fast diagnosis time.

### Performance of the microfluidic microplate for *Pf*LDH immunoassay

**Fig. 2:**
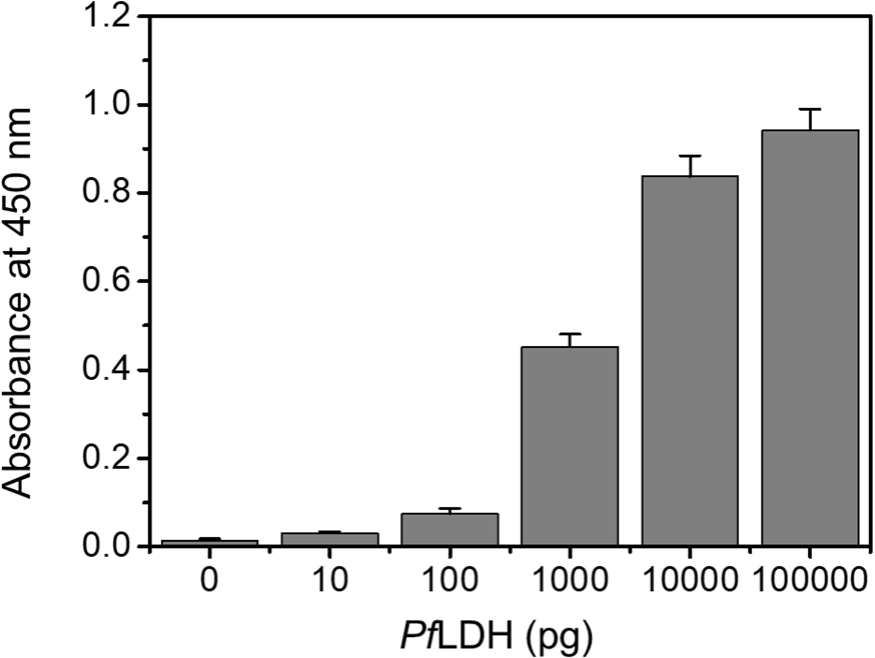
Diagnosis of *Pf*LDH using the ELISA. The optical density were measured at 450 nm wavelength with 0 to 100 ng/100 μL (n = 3)

**Fig. 3:**
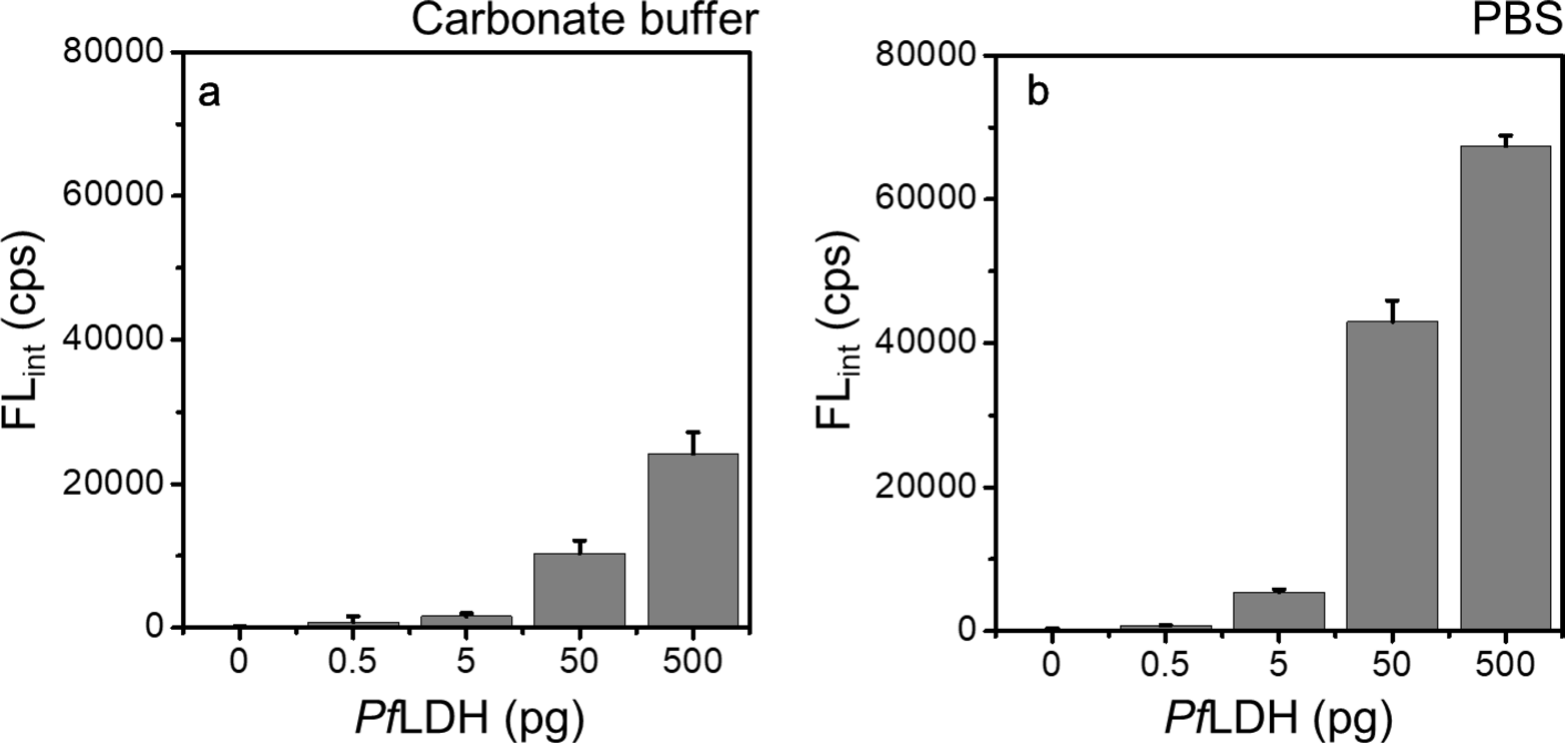
Optimization of the Cap-Ab adsorption. The Cap-Ab coating efficiency was compared two coating buffers. To detect the *Pf*LDH in the microfluidic microplate, the Cap-Ab was prepared in **a** carbonate-bicarbonate buffer (pH 9.6) and **b** PBS (pH 7.4). The chemiluminescence were measured by BioTek multimode reader (Excitation: 530 nm, Emission: 590 nm, n = 3)

To optimize the microfluidic microplate-based immunoassay for *Pf*LDH, we selected an antibody pair through a sandwich ELISA (data not shown). Based on the results of the ELISA, monoclonal mouse *Pf*LDH antibody was selected as the capture antibody (Cap-Ab), and polyclonal rabbit *Pf*LDH antibody was selected as the primary antibody (1st-Ab). In this condition, we confirmed that *Pf*LDH was diagnosed up to 0.1 pg/µL (10 pg in 100 μL) by ELISA (Fig. 2). To use selected antibody pairs in the microfluidic microplate, the optimization of Cap-Ab adsorption was significantly important to the whole immunoassay process. We compared the diagnosis efficiency in carbonate-bicarbonate buffer (pH 9.6, Fig. 3a) and phosphate-buffered saline (PBS, pH 7.4, Fig. 3b) to optimize Cap-Ab adsorption. In general, carbonate-bicarbonate buffer was used as a coating buffer in ELISA because the high pH was attributed to the better dissolution of proteins into the buffer and improved adsorption to the positive charged plate. However, Cap-Ab in PBS showed better diagnosis efficiency than Cap-Ab in carbonate-bicarbonate buffer (Fig. 3). This indicates that PBS (pH 7.4) was appropriate substance to adsorb Cap-Ab on the microfluidic microplate wall.

**Fig. 4:**
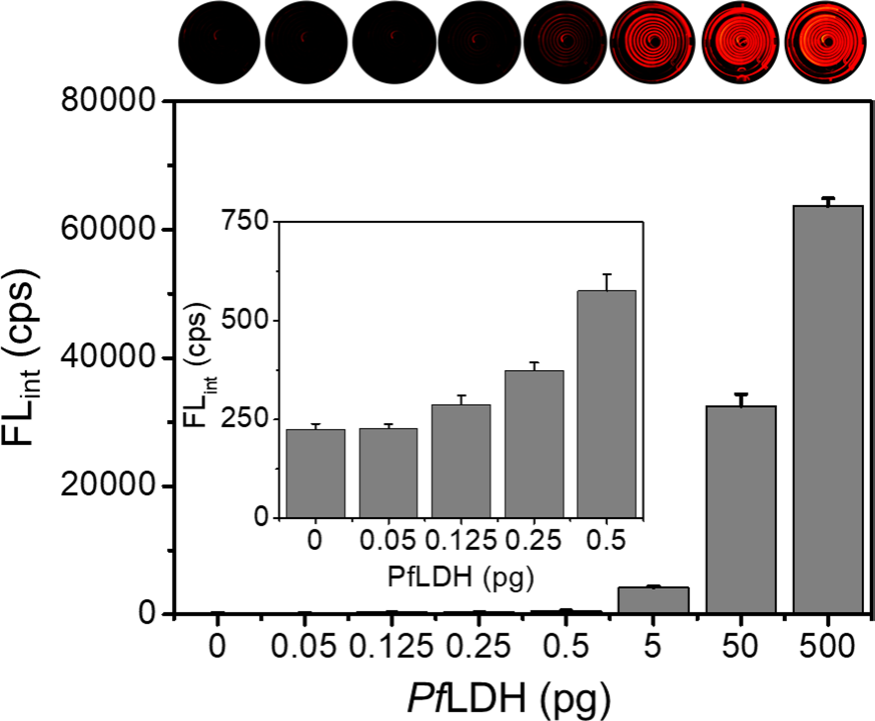
Diagnosis of *Pf*LDH using the microfluidic microplate-based immunoassay. Chemiluminescence intensity (n = 3) and chemiluminescence images of the microfluidic microplate to the detection of *Pf*LDH

**Fig. 5:**
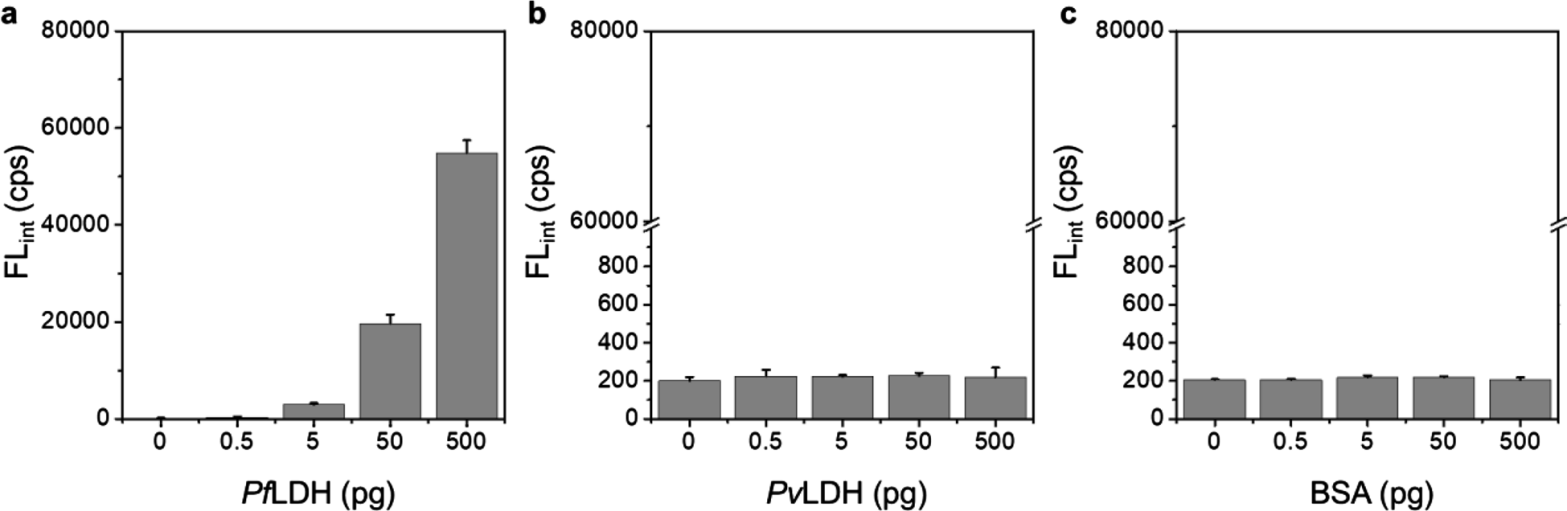
Selectivity and specificity of microfluidic microplate-based immunoassay for detection of *Pf*LDH. Diagnosis of **a***Pf*LDH, **b***Pv*LDH, and **c** BSA in PBS-based blocking solution (n = 3)

To evaluate the *Pf*LDH diagnostics capability in the microfluidic microplate, we diagnosed *Pf*LDH in the range from 0.05 to 500 pg, including the negative control (*Pf*LDH 0 pg). As shown in Fig. 4, *Pf*LDH showed a limit of detection (LOD) of 0.025 pg/µL (0.125 pg in 5 μL), and the diagnosis of *Pf*LDH was visually confirmed through the chemiluminescence image. The LOD was calculated as the minimum detectable signal (FL_*Pf*LDH=0_ + 3SD_*Pf*LDH=0_). Additionally, we compared *Pf*LDH with *Plasmodium vivax* lactate dehydrogenase (*Pv*LDH) and BSA for selectivity and specificity at concentrations of 0, 0.5, 5, 50, and 500 pg/5 µL. As shown in Fig. 5, this platform showed high selectivity and specificity for *Pf*LDH. Based on the results of experiments, it was clearly shown that *Pf*LDH can be ultrafast and precise in our sensing platform.

**Fig. 6:**
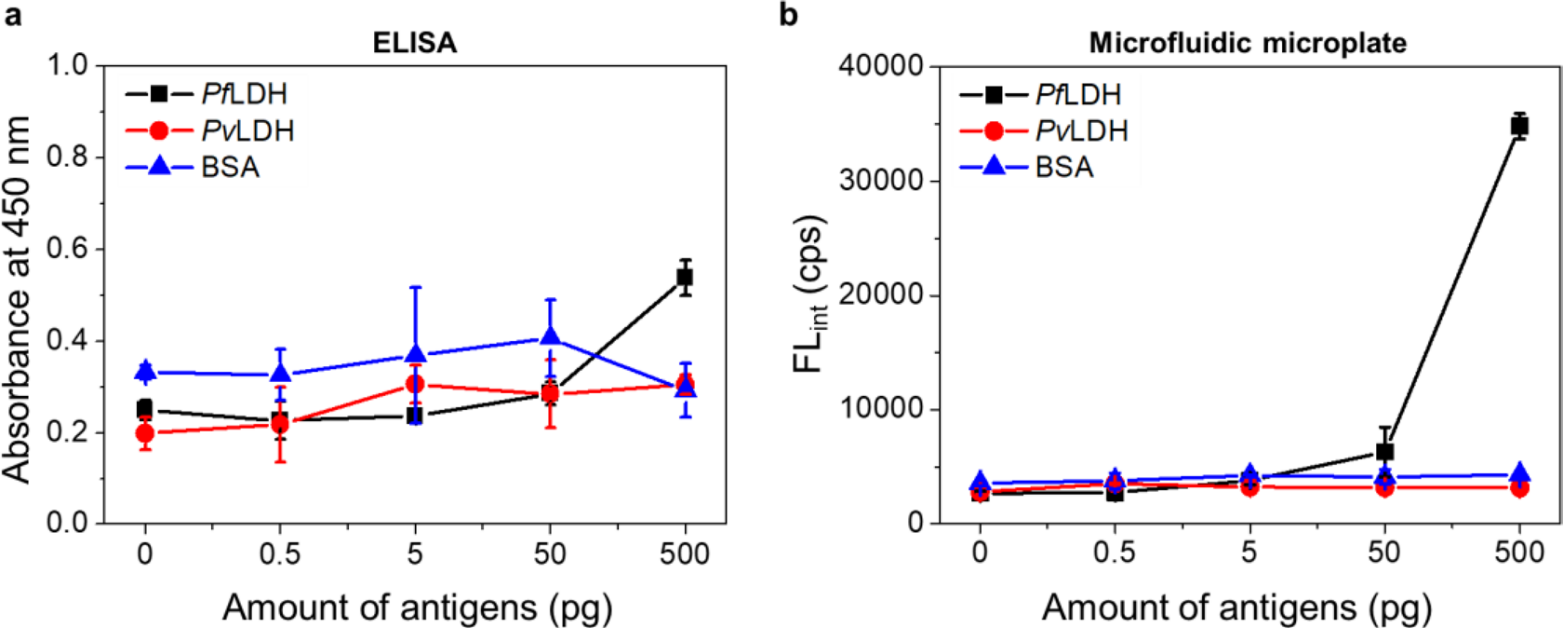
Diagnosis of *Pf*LDH, *Pv*LDH, and BSA in human serum using **a** ELISA and **b** microfluidic microplate (n = 3). All antigens were prepared in the range of 0–500 pg in human serum

To confirm its applicability in clinical diagnosis, we performed the diagnosis of *Pf*LDH in human serum. *Pf*LDH, *Pv*LDH, and BSA were prepared in commercial human serum at concentrations of 0, 0.5, 5, 50, and 500 pg/5 µL. Figure 6a shows the diagnosis results of the *Pf*LDH in human serum using ELISA. The results show that the non-specific binding is high, even in the absence of *Pf*LDH. It means that *Pf*LDH in human serum can be detected by ELISA but is not suitable for highly sensitive diagnosis due to its high non-specific binding. In general, one of the drawbacks of ELISA is the false-positive result due to non-specific binding in the presence of various proteins, such as human serum, it must be improved for application in clinical diagnosis [28]. In contrast to the ELISA results, Fig. 6b shows that *Pf*LDH in human serum can be diagnosed up to 1 pg/µL (5 pg in 5 μL) with high selectivity and specificity using microfluidic microplate. This LOD is an ideal sensitivity at which to adapt the clinical application because *Pf*LDH-infected patients typically show *Pf*LDH plasma levels of approximately 3–15 pg/μL [13, 29]. Recently, Tonigold et al. demonstrated that the conditions of antibody adsorption (i.e., pH and isoelectric point of antibody) were significantly affected by the orientation of the antibody [30]. They validated that adsorbed antibodies on the polystyrene (PS) beads showed superior targeting properties compared with covalently coupled antibodies with the PS beads. The microfluidic microplate is also composed of PS; therefore, the Cap-Ab, which is well oriented on the microfluidic channel, led to the high sensing capability of *Pf*LDH, including *Pf*LDH spiked in human serum. Additionally, the large surface area and capillary forces of the microfluidic channel also contributed to the sensing performance. Based on the results of *Pf*LDH diagnosis in human serum, we expect that our immunoassay platform can be widely used for the clinical diagnosis of infectious diseases such as malaria.

### Diagnostics capability of the microfluidic microplate-based immunoassay

Table 1 summarizes and compares recent studies on the diagnosis of *Pf*LDH. Recently, many studies have developed high-sensitivity, high-selectivity, and cost-effective diagnostics based on aptamers or nanoparticles [31–35]. Nanoparticles, such as novel-metal nanoparticles and magnetic nanoparticles, are applied for lateral flow immunoassay or combined with aptamers to improved diagnostic performance. However, these sensors require the complicated surface functionalization and stabilization of nanoparticles or require a relatively large amount of sample volume. On the other hand, the microfluidic microplate-based immunoassay improved the conventional 96-well plate to a microfluidic channel, significantly reducing the time and amount of reagents required for the diagnosis and showing that high-sensitive diagnosis to the *Pf*LDH. Based on the results, the microfluidic microplate-based immunoassay shows that it not only brings cost-effective for antibody use but also provides easier, simpler, faster, and superior sensitive diagnosis than recently developed sensors.

**Table 1:** Comparison of recent *Pf*LDH detection studies

Diagnosis	Target	Bio-recognition	Output	LOD	Volume	References
Microfluidic microplate	*Pf*LDH in buffer	Antibody	Fluorescence intensity	0.025 pg/μL	5 μL	This study
Microfluidic microplate	*Pf*LDH in human serum	Antibody	Fluorescence intensity	1 pg/μL	5 μL	This study
ELISA	*Pf*LDH in buffer	Antibody	Absorbance	0.1 pg/μL	100 μL	This study
Lateral flow immunoassay	*Pf*LDH in buffer	Antibody	Colorimetric assay	10 pg/μL	30 μL	[31]
AuNPs based aptasensor	*Pf*LDH in lysed RBCs^a^ solution (Mimic real sample)	Aptamer	Colorimetric assay	38 pg/μL	10 μL	[32]
MNP-Qdot aptasensor	*Pf*LDH in buffer	Aptamer	Fluorescence intensity	0.0066 pg/μL	100 μL	[33]
Aptamer-tethered enzyme capture (APTEC) assay	*Pf*LDH in buffer	Aptamer	Colorimetric assay	4.9 pg/μL	10 μL	[34]
Aptamer-tethered enzyme capture (APTEC) assay	*Pf*LDH in serum	Aptamer	Colorimetric assay	50 pg/μL	40 μL	[35]

^a^*RBC* red blood cells

## Conclusions

In conclusion, we demonstrated that simple, ultrafast, and accurate diagnosis of *Pf*LDH using microfluidic microplates is possible. We reduced the amount of reagent required for diagnosis to 5 μL and reduced the diagnosis time to 90 min. The microfluidic microplate-based sensing platform provides a user-friendly method and a cost-effective approach for an immunoassay-based method for *Pf*LDH diagnosis. Our sensing platform with optimized antibody pairs of capture and detection antibodies showed highly sensitive, selective, and specific diagnosis of *Pf*LDH. We expect that microfluidic microplates will be used for malaria diagnosis rapidly and accurately in hospitals.

## Experimental section

### Materials

The microfluidic microplate plate (Optimiser™ microplate), holder, and absorbent pad were provided from MiCo BioMed Co., Ltd. (Seoul, Korea). Mouse monoclonal *Pf*LDH antibody was purchased from Fapon Biotech (BRCMALS212, Guangdong, China), and rabbit polyclonal *Pf*LDH antibody was purchased from LifeSpan BioScience, Inc. (LS-C488775, Seattle, USA). *Pf*LDH and *Pv*LDH were provided by BioNano Health Guard Research Center (H-GUARD). Polyclonal anti-rabbit HRP-tagged antibody (HRP-Ab) was obtained from Cell Signaling Technology, Inc. (Danvers, USA). Phosphate-buffered saline (PBS), blocking solution (StartingBlock™) and HRP substrate kit (QuantaRed™ Enhanced Chemifluorescent HRP substrate kit) were purchased from Thermo Fisher Scientific Ltd. (Waltham, USA). Bovine serum albumin (BSA) and sodium carbonate were purchased from Sigma-Aldrich (Louis, USA). Human serum (from human male AB plasma, USA origin, sterile-filtered) was purchased from Sigma-Aldrich (Louis, USA).

### Expression and purification of *Pf*LDH

As malaria biomarkers, *Pf*LDH and *Pv*LDH were prepared for malaria diagnostics. In order to clone *Pf*LDH and *Pv*LDH, the full length gene encoding for the LDH was amplified via the polymerase chain reaction (PCR). The PCR product was inserted into the pET 21a vector using to generate pET-*Pf*LDH and pET-*Pv*LDH. The gene was all verified by DNA sequencing, and transformed into the expression host, *E. coli* BL21 (DE3) (Stratagene, CA) for the expression of the recombinant proteins. The transformed cells were grown at 37 °C with shaking to an OD600 of 0.6. The cells were induced with 1 mM isopropyl-2-*D*-thiogalactopyranoside (IPTG, GibcoBRL, MD), and grown for an additional 14 h at 25 °C. The cells were then harvested and disrupted via sonication, after which the soluble and insoluble fractions were separated by centrifugation. The soluble fractions were loaded onto a IDA-miniexcellose affinity column (Bioprogen Co., Republic of Korea) and washed three times with equilibration buffer (50 mM Tris–Cl, 0.5 N NaCl, pH 8.0), respectively. The recombinant proteins were then eluted with 0.5 M imidazole in the same buffer (50 mM Tris–Cl, 0.5 N NaCl, pH 8.0), and dialyzed against phosphate-buffered saline (PBS, pH7.4). The purified *Pf*LDH and *Pv*LDH were resolved on 12% Sodium dodecyl sulfate–polyacrylamide gel electrophoresis (SDS-PAGE) and the gels were stained with Coomassie’s Brilliant Blue R250. Protein concentrations were determined by the Bradford method, using bovine serum albumin as a standard.

### Validation of antibody pairs using ELISA

To select the antibody pairs for detection of *Pf*LDH, Cap-Ab and 1st-Ab were selected by ELISA. As a Cap-Ab, mouse monoclonal *Pf*LDH antibody (200 μL, 2 μg/mL) dissolved in sodium bicarbonate 1 M (pH 9.6), is incubated in a 96-well plate (Corning^®^ 96 Well EIA/RIA Assay Microplate) at 4 °C for overnight. After the reaction, BSA 1% in PBS buffer (100 μL) was added to the wells and reacted at 37 °C for 30 min. After the removal of BSA solution, *Pf*LDH (100 μL dissolved in PBS) was added to the wells in a concentration range of 1 μg/mL to 100 μg/mL and reacted at 37 °C for 120 min. After *Pf*LDH treatment, rabbit polyclonal *Pf*LDH antibody as a 1st-Ab (100 μL, 1/2000 dilution) was immediately added onto the *Pf*LDH solution. After 120 min incubation at 37 °C, the wells were washed 2 times with PBS buffer. Then, HRP-Ab was added on the wells and reacted at 37 °C for 120 min. Once again, the wells were washed 4 times with PBS buffer. Finally, TMB substrate reagent (BD Biosciences, USA) composed of TMB and hydrogen peroxide are mixed at a ratio of 1: 1, and 100 μL was added each well. After 5 min, stop solution was added onto the wells and measured absorbance at 450 nm in microplate reader (Multiskan™ FC Microplate Photometer, Thermo Fisher Scientific, USA).

### Immunoassay of *Pf*LDH using the microfluidic microplate

The immunoassay of *Pf*LDH in the microfluidic microplate was performed in the same order as conventional ELISA. Cap-Ab (5 μL, 10 μg/mL) dispersed in coating buffer (PBS and carbonate-bicarbonate buffer) was first immobilized on the microfluidic channel for 10 min. Five microliters of blocking solution were added to the channels and incubated for 10 min. Next, *Pf*LDH (each 5 μL, 0–100 ng/mL) and 1st-Ab (1/100 dilution) were treated and incubated for 10 min in order. The microplates were then incubated with 5 μL PBS for 10 min to remove unbound substances and were treated with HRP-tagged Ab (1/2500 dilution) for 10 min. Then, all channels were washed with PBS (30 μL) twice. After washing, Quantared™ Enhanced Chemifluorescent HRP Substrate (Enhancer solution: Stable peroxide solution: ADHP = 50:50:1) was added to the channel for chemiluminescence. After 10 min, the chemiluminescent signal was measured using a BioTek multimode reader (Cytation5, USA). Additionally, the chemiluminescence image was observed by stereomicroscope (SMZ18, Nikon, Japan) with a fluorescence filter (excitation 530 nm). To confirm the diagnostic ability of *Pf*LDH in human serum, *Pf*LDH, *Pv*LDH and BSA were prepared from human serum. Three antigens were prepared in PBS 10 times higher than the target concentration before diagnosis. These antigens were then spiked in human serum at a volume ratio of 1:9. The immunoassay procedure was the same as described above.

## Data Availability

All data generated or analyzed during this study are included in this published article.

## Abbreviations

WHO: World Health Organization
*P. falciparum*: *Plasmodium falciparum*
*P. vivax*: *Plasmodium vivax*
LDH: Lactate dehydrogenase
HRP2: Histidine rich protein2
*Pf*LDH: *Plasmodium falciparum* lactate dehydrogenase
*Pv*LDH: *Plasmodium vivax* lactate dehydrogenase
RDTs: Rapid diagnostic tests
ELISA: Enzyme-linked immunosorbent assay
TMB: 3,3′,5,5′-tetramethylbenzidine
ADHD: 10-acetyl-3,7-dihydroxyphenoxazine
HRP: Horseradish peroxidase
Cap-Ab: Capture antibody
1st-Ab: Primary antibody
HRP-Ab: HRP-tagged antibody
PBS: Phosphate-buffered saline
BSA: Bovine serum albumin
LOD: Limit of detection
PCR: Polymerase chain reaction
SDS-PAGE: Sodium dodecyl sulfate–polyacrylamide gel electrophoresis
APTEC: Aptamer-tethered enzyme capture
H-GUARD: BioNano Health Guard Research Center
